# Supplementary material for: High throughput parameter estimation and uncertainty analysis applied to the production of mycoprotein from synthetic lignocellulosic hydrolysates
Source: Curr Res Food Sci. 2024 Oct 28;9:100908. doi: 10.1016/j.crfs.2024.100908 (PMC11565039; doi:10.1016/j.crfs.2024.100908)
Supplement: Multimedia component 1 [file mmc1.docx]

**Supplementary Materials**

High Throughput Parameter Estimation and Uncertainty Analysis Applied to the Production of Mycoprotein from Synthetic Lignocellulosic Hydrolysates

Mason Banks ^a^, Mark Taylor ^b^, Miao Guo ^a, *^

^a^ Department of Engineering, Faculty of Natural Mathematical & Engineering Sciences, King’s College London, Strand, London, WC2R 2LS, United Kingdom

^b^ Fermentation Lead, Marlow Ingredients, Nelson Ave, Billingham, North Yorkshire, TS23 4HA, United Kingdom

*Corresponding author: [miao.guo@kcl.ac.uk](mailto:miao.guo@kcl.ac.uk)

Table of Contents

[A. Raw time series data 1](#_Toc180503326)

[i. 2:1 glucose-xylose initial condition 1](#_Toc180503327)

[ii. 1:1 glucose-xylose initial condition 3](#_Toc180503328)

[B. Optical density at 600nm to cell dry weight correlation 4](#_Toc180503329)

[i. Data summary 4](#_Toc180503330)

[ii. Example calculations 5](#_Toc180503331)

[iii. Calibration curve 6](#_Toc180503332)

[C. Differential evolution (DE) algorithm hyperparameter selection 7](#_Toc180503333)

[D. Pellicle formation in plate wells 9](#_Toc180503334)

[E. Parameter correlations and p-values 10](#_Toc180503335)

[F. Contour plot of objective function with covariation of $\boldsymbol{Kc}\boldsymbol{1}$ and $\boldsymbol{Kc}\boldsymbol{2}$ 11](#_Toc180503336)

# **Raw time series data**

## 2:1 glucose-xylose initial condition

**Table 1**. OD600 and substrate concentration data for batch microlitre fermentation (G2X1 condition). Undetectable concentrations are assigned an arbitrarily low value of 0.001 g/L and indicated by italic font.

| Time (h) | OD600 (-) | [Glucose] (g/L) | [Xylose] (g/L) |
| --- | --- | --- | --- |
| 24.000 | 0.144 | 20.198 | 10.272 |
| 24.000 | 0.139 | 20.385 | 10.876 |
| 24.000 | 0.140 | 20.358 | 10.367 |
| 32.333 | 0.257 | 17.018 | 8.981 |
| 32.333 | 0.277 | 19.677 | 10.226 |
| 32.333 | 0.234 | 20.140 | 10.448 |
| 49.333 | 0.535 | 14.328 | 9.048 |
| 49.333 | 0.446 | 16.502 | 9.950 |
| 49.333 | 0.433 | 18.731 | 11.044 |
| 51.000 | 0.469 | 18.513 | 10.948 |
| 51.000 | 0.380 | 18.987 | 11.062 |
| 51.000 | 0.381 | 17.310 | 10.166 |
| 51.000 | 0.391 | 18.480 | 10.850 |
| 51.000 | 0.450 | 18.776 | 11.028 |
| 51.000 | 0.544 | 17.385 | 10.533 |
| 53.333 | 0.548 | 17.885 | 10.998 |
| 53.333 | 0.531 | 18.204 | 11.065 |
| 53.333 | 0.457 | 16.034 | 9.906 |
| 56.333 | 0.742 | 16.788 | 11.099 |
| 56.333 | 0.585 | 10.870 | 7.170 |
| 56.333 | 0.662 | 16.260 | 10.519 |
| 59.667 | 1.005 | 15.566 | 11.176 |
| 59.667 | 0.657 | 16.274 | 11.346 |
| 59.667 | 0.895 | 15.035 | 10.645 |
| 62.333 | 1.268 | 13.552 | 11.308 |
| 62.333 | 0.903 | 15.481 | 11.365 |
| 62.333 | 1.068 | 13.837 | 10.860 |
| 65.000 | 1.222 | 11.483 | 11.372 |
| 65.000 | 1.223 | 13.187 | 11.551 |
| 65.000 | 1.362 | 9.003 | 10.847 |
| 69.333 | 1.657 | 1.583 | 4.679 |
| 69.333 | 1.651 | 2.741 | 10.571 |
| 69.333 | 1.809 | 1.344 | 5.284 |
| 73.667 | 2.014 | *0.001* | 9.228 |
| 73.667 | 2.252 | *0.001* | 8.694 |
| 73.667 | 2.019 | *0.001* | 8.611 |
| 76.667 | 2.207 | *0.001* | 6.169 |
| 76.667 | 2.139 | *0.001* | 5.612 |
| 76.667 | 2.168 | *0.001* | 7.032 |
| 80.333 | 2.271 | *0.001* | 1.118 |
| 80.333 | 2.261 | *0.001* | 5.204 |
| 80.333 | 2.146 | *0.001* | 4.373 |
| 84.333 | 2.327 | *0.001* | 1.241 |
| 84.333 | 2.313 | *0.001* | 0.219 |
| 84.333 | 2.366 | *0.001* | 1.856 |
| 88.000 | 2.257 | *0.001* | 0.202 |
| 88.000 | 2.279 | *0.001* | 0.356 |
| 88.000 | 2.316 | *0.001* | 0.175 |
| 95.667 | 2.556 | *0.001* | 0.067 |
| 95.667 | 2.378 | *0.001* | 0.088 |
| 95.667 | 2.287 | *0.001* | 0.068 |
| 121.333 | 2.495 | *0.001* | 0.034 |
| 121.333 | 2.465 | *0.001* | 0.036 |
| 121.333 | 2.504 | *0.001* | 0.019 |
| 121.333 | 2.559 | *0.001* | 0.035 |
| 121.333 | 2.555 | *0.001* | 0.019 |
| 121.333 | 2.517 | *0.001* | 0.017 |
| 121.333 | 2.542 | *0.001* | 0.004 |
| 121.333 | 2.651 | *0.001* | 0.028 |
| 121.333 | 2.667 | *0.001* | 0.030 |

## 1:1 glucose-xylose initial condition

**Table 2**. OD600 and substrate concentration data for batch microlitre fermentation (G1X1 condition). Undetectable concentrations are assigned an arbitrarily low value of 0.001 g/L and indicated by italic font.

| Time (h) | OD600 (-) | [Glucose] (g/L) | [Xylose] (g/L) |
| --- | --- | --- | --- |
| 24.000 | 0.234 | 15.233 | 15.964 |
| 24.000 | 0.204 | 15.232 | 15.974 |
| 24.000 | 0.164 | 15.153 | 15.873 |
| 32.333 | 0.204 | 14.675 | 15.918 |
| 32.333 | 0.239 | 14.650 | 15.996 |
| 32.333 | 0.360 | 14.726 | 16.109 |
| 49.333 | 0.428 | 12.395 | 16.083 |
| 49.333 | 0.446 | 12.404 | 16.162 |
| 49.333 | 0.523 | 12.214 | 16.112 |
| 51.000 | 0.395 | 13.502 | 16.262 |
| 51.000 | 0.443 | 12.985 | 16.100 |
| 51.000 | 0.378 | 12.944 | 15.968 |
| 51.000 | 0.364 | 13.320 | 16.140 |
| 51.000 | 0.441 | 12.855 | 15.772 |
| 51.000 | 0.593 | 12.786 | 15.898 |
| 53.333 | 0.504 | 12.438 | 15.910 |
| 53.333 | 0.465 | 12.387 | 15.830 |
| 53.333 | 0.495 | 12.369 | 15.897 |
| 56.333 | 0.673 | 11.654 | 16.383 |
| 56.333 | 0.578 | 11.663 | 15.949 |
| 56.333 | 0.593 | 10.991 | 16.036 |
| 59.667 | 0.767 | 10.963 | 16.202 |
| 59.667 | 1.014 | 10.116 | 16.352 |
| 59.667 | 0.899 | 10.508 | 16.409 |
| 62.333 | 1.137 | 8.462 | 16.510 |
| 62.333 | 1.188 | 7.715 | 16.195 |
| 62.333 | 1.075 | 8.077 | 16.381 |
| 65.000 | 1.030 | 8.337 | 16.703 |
| 65.000 | 1.243 | 7.447 | 16.468 |
| 65.000 | 1.426 | 4.760 | 16.573 |
| 69.333 | 1.759 | 0.606 | 15.364 |
| 69.333 | 1.573 | 0.700 | 9.001 |
| 69.333 | 2.058 | *0.001* | 13.788 |
| 73.667 | 2.072 | *0.001* | 13.127 |
| 73.667 | 2.083 | *0.001* | 13.449 |
| 73.667 | 2.194 | *0.001* | 12.862 |
| 76.667 | 2.044 | *0.001* | 9.253 |
| 76.667 | 2.321 | *0.001* | 11.372 |
| 76.667 | 2.170 | *0.001* | 10.106 |
| 80.333 | 2.134 | *0.001* | 5.532 |
| 80.333 | 2.271 | *0.001* | 8.331 |
| 80.333 | 2.328 | *0.001* | 7.282 |
| 84.333 | 2.322 | *0.001* | 4.292 |
| 84.333 | 2.304 | *0.001* | 3.899 |
| 84.333 | 2.267 | *0.001* | 3.705 |
| 88.000 | 2.573 | *0.001* | 0.087 |
| 88.000 | 2.467 | *0.001* | 0.231 |
| 88.000 | 2.410 | *0.001* | 0.882 |
| 95.667 | 2.299 | *0.001* | 0.124 |
| 95.667 | 2.418 | *0.001* | 0.090 |
| 95.667 | 2.679 | *0.001* | 0.036 |
| 121.333 | 2.513 | *0.001* | 0.020 |
| 121.333 | 2.665 | *0.001* | 0.057 |
| 121.333 | 2.575 | *0.001* | 0.041 |
| 121.333 | 2.512 | *0.001* | 0.020 |
| 121.333 | 2.724 | *0.001* | 0.021 |
| 121.333 | 2.567 | *0.001* | 0.034 |
| 121.333 | 2.630 | *0.001* | 0.007 |
| 121.333 | 2.632 | *0.001* | 0.029 |
| 121.333 | 2.649 | *0.001* | 0.036 |

# **Optical density at 600nm to cell dry weight correlation**

## Data summary

**Table 3**. Summary of experimental data used for determination of cell dry weight - optical density correlation. Includes the mean optical density at 600 nm (OD600) and its absolute standard deviation (SD), total dry mass measured, culture volume, and calculated cell dry weight. The uncertainties for total dry mass, volume, and cell dry weight are provided in parentheses.

| Time point(s) (h) | No. Samples | OD600 (-) | | Total Dry Mass (±0.1 mg) | Volume (±0.8 μL) | Cell Dry Weight (±0.1 g/L) |
| --- | --- | --- | --- | --- | --- | --- |
|  |  | Mean | \|SD\| |  |  |  |
| 51.0 | 12 | 0.436 | 0.071 | 0.5 | 150.0 | 0.3 |
| 53.3, 56.6 | 12 | 0.569 | 0.088 | 1.6 | 150.0 | 0.9 |
| 59.7, 62.3 | 12 | 0.990 | 0.175 | 3.3 | 150.0 | 1.8 |
| 65.0 | 6 | 1.251 | 0.137 | 2.1 | 150.0 | 2.3 |
| 69.3 | 6 | 1.751 | 0.172 | 4.1 | 150.0 | 4.6 |
| 73.7 | 6 | 2.106 | 0.097 | 5.0 | 150.0 | 5.6 |
| 76.7 | 5 | 2.146 | 0.090 | 4.6 | 150.0 | 6.1 |
| 84.3 | 6 | 2.317 | 0.032 | 8.1 | 150.0 | 9.0 |
| 95.7 | 6 | 2.436 | 0.153 | 9.1 | 150.0 | 10.1 |
| 121.3 | 6 | 2.501 | 0.019 | 8.4 | 150.0 | 9.3 |
| 121.3 | 5 | 2.560 | 0.012 | 6.3 | 150.0 | 8.4 |
| 121.3 | 7 | 2.660 | 0.032 | 11.2 | 150.0 | 10.7 |

## Example calculations

*Mean OD600:*

$\bar{OD600}=\frac{\left( 0.469 + 0.38 + 0.381 + 0.395 + 0.443 + 0.378 + 0.391 + 0.45 + 0.544 + 0.364 + 0.441 + 0.593 \right)}{12}=\frac{5.229}{12}=0.43575\approx0.436 (3dp)$

*Standard Deviation of OD600:*

$$\sigma_{OD600}= \sqrt{\frac{\sum\left( {OD600}_{i}-\bar{OD600} \right)^{2}}{n-1}}$$

$$=\sqrt{\frac{\left( 0.469-0.43575 \right)^{2}+\left( 0.380-0.43575 \right)^{2}+\ldots+ \left( 0.593-0.43575 \right)^{2}}{11}}=\sqrt{0.00509}\approx\pm0.071 (3dp)$$

*Calculation of CDW:*

$$CDW = Total Dry Mass / (No. of samples \times Volume)$$

$$=0.5 mg / (12 \times0.150 mL) = 0.5 / 1.8 = 0.2778 g/L \approx0.3 g/L (1dp)$$

*Relative Uncertainty in Total Dry Mass:*

$$\frac{\Delta M}{M}=\frac{\sqrt{{(0.08 mg)}^{2}{+(0.06 mg)}^{2}}}{0.5 mg}=\frac{\sqrt{{0.01 mg}^{2}}}{0.5 mg}=\frac{\pm0.1 mg}{0.5 mg}=\pm0.2$$

*Relative Uncertainty in Volume:*

$$\frac{\Delta V}{V}=\frac{\sqrt{{(0.8 \mu L)}^{2}{+(0.2 \mu L)}^{2}}}{150 \mu L}=\frac{\sqrt{{0.68 \mu L}^{2}}}{150 \mu L}=\frac{\pm0.82 \mu L}{150 \mu L}=\pm0.0053$$

*Combined Uncertainty in CDW:*

$$\left( \frac{\Delta CDW}{CDW} \right)^{2}= \left( \frac{\Delta M}{M} \right)^{2} + \left( \frac{\Delta V}{V} \right)^{2}$$

$${=(0.2)}^{2}{+ (0.0053)}^{2}=0.040+0.00003=0.04003$$

$$\Rightarrow\Delta CDW= 0.3 \times\sqrt{0.04003}=0.3 \times0.2001=0.06003 \approx\pm0.1 g/L (1 dp)$$

## Calibration curve


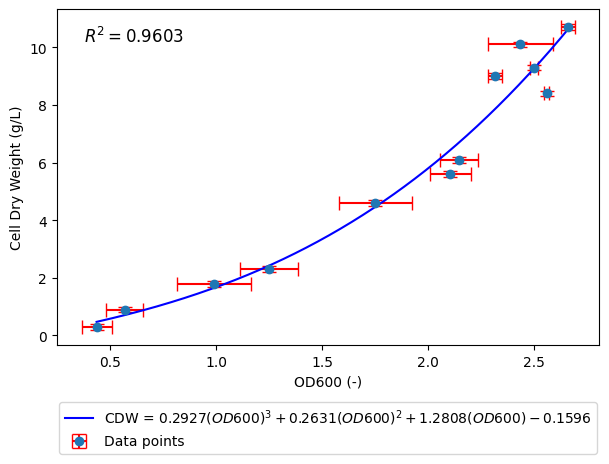


**Fig. 1**. Calibration Curve. Data points (blue dots with red bars) representing mean and uncertainty of CDW and corresponding mean and standard deviation of OD600 for the time-series fermentation experiments. The cubic regression function with R^2^ = 0.9603 is plotted (blue line).

# **Differential evolution (DE) algorithm hyperparameter selection**

A comprehensive evaluation of the DE algorithm the was performed to determine the most effective approach to estimating parameters of the mixed substrate fermentation model. The primary goal was to identify the combination of hyperparameters that not only achieves high accuracy in parameter estimation but also demonstrates robustness and efficiency in terms of convergence to the global optimum. Each method was assessed based on the objective function value and the number of iterations required to achieve convergence, and each test was performed utilising the same data set (mean-aggregated G1X1 time series).

The experiments showed that while all tested combinations could achieve objective function values close to zero (Fig. 2), indicating a high level of accuracy, their efficiencies differed significantly. To further validate the robustness of the DE algorithm, we conducted five replicate runs with varying random seeds and observed a high degree of consistency in the parameter estimates, reinforcing the algorithm's reliability in locating the likely global optimum.

Based on these findings, we selected the DE algorithm with a population size of 50, mutation factor range of (0.5, 1), and recombination rate of 0.7. This setting not only achieved convergence to the global optimum with high consistency but also required significantly fewer iterations compared to other configurations (Fig. 3). The consistent performance of DE across different runs and its ability to efficiently converge to the global optimum underscore its utility in complex parameter estimation tasks, particularly in the context of the highly non-linear fermentation model under study.

| 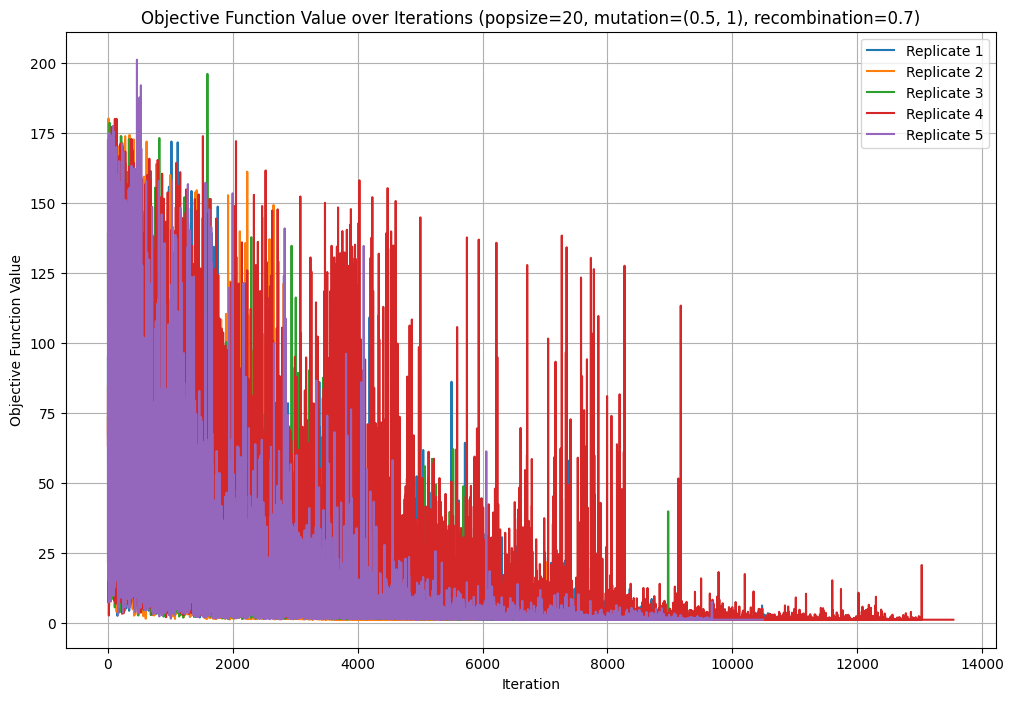 | 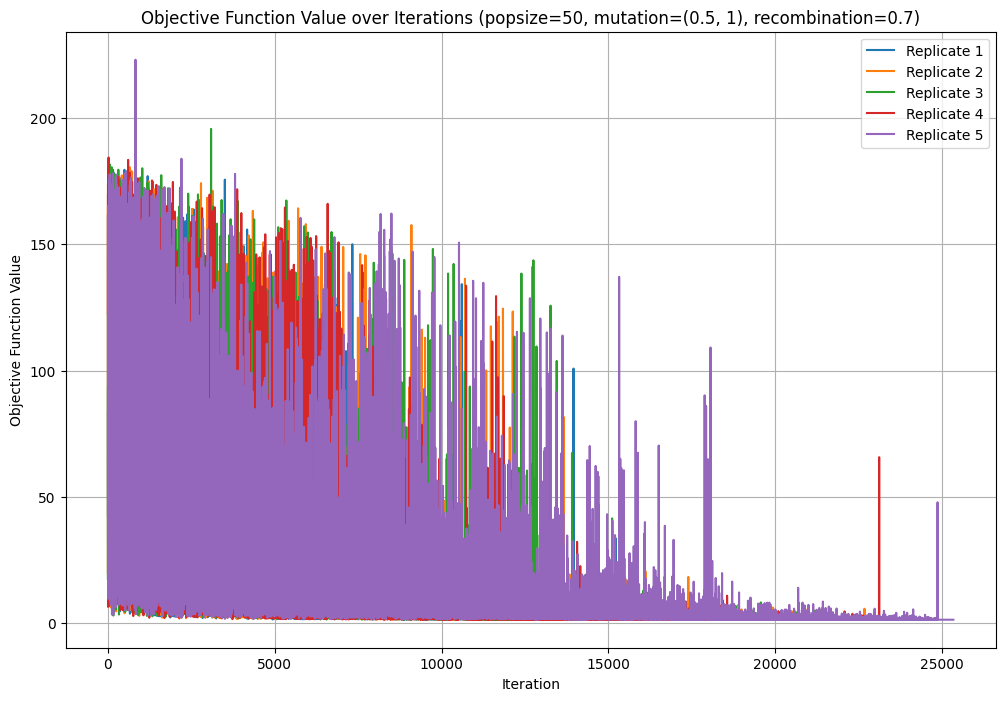 |
| --- | --- |
| 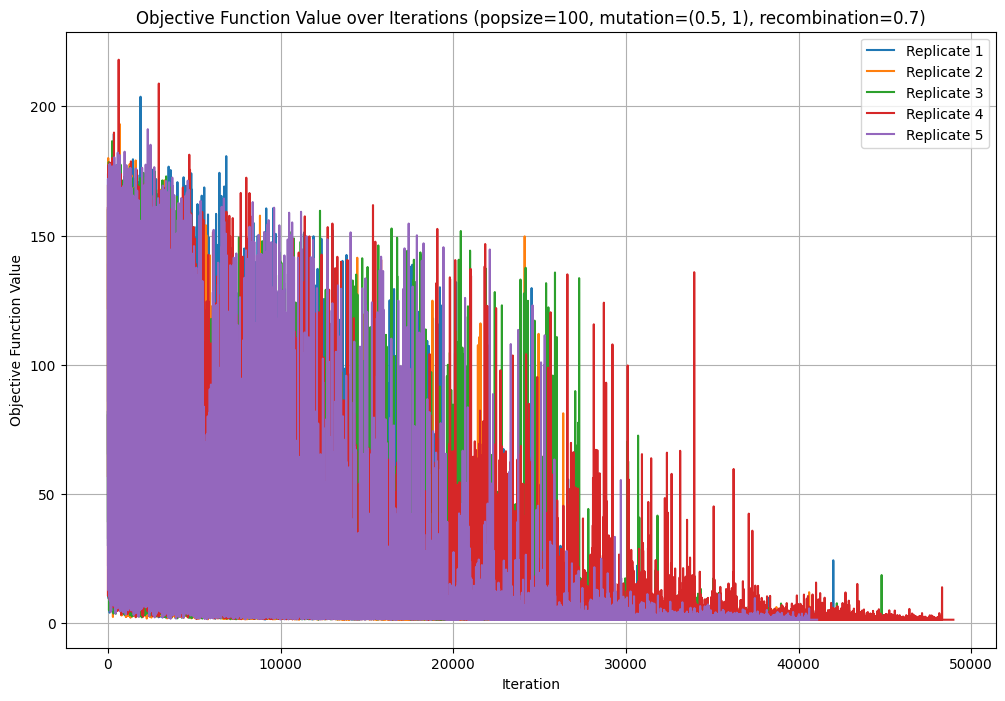 | 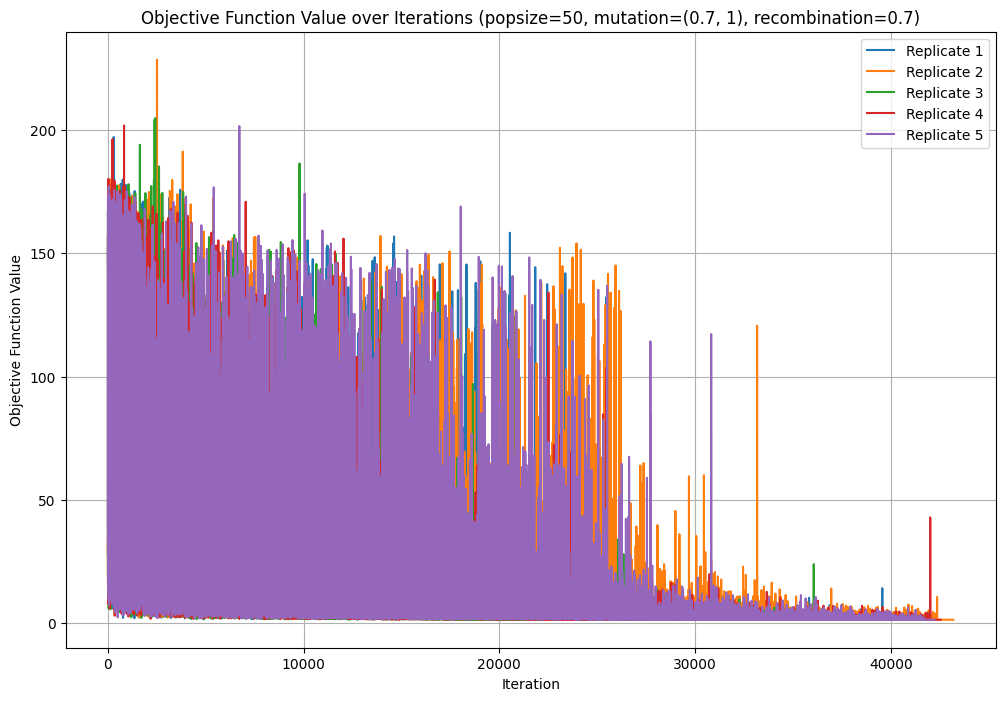 |
| 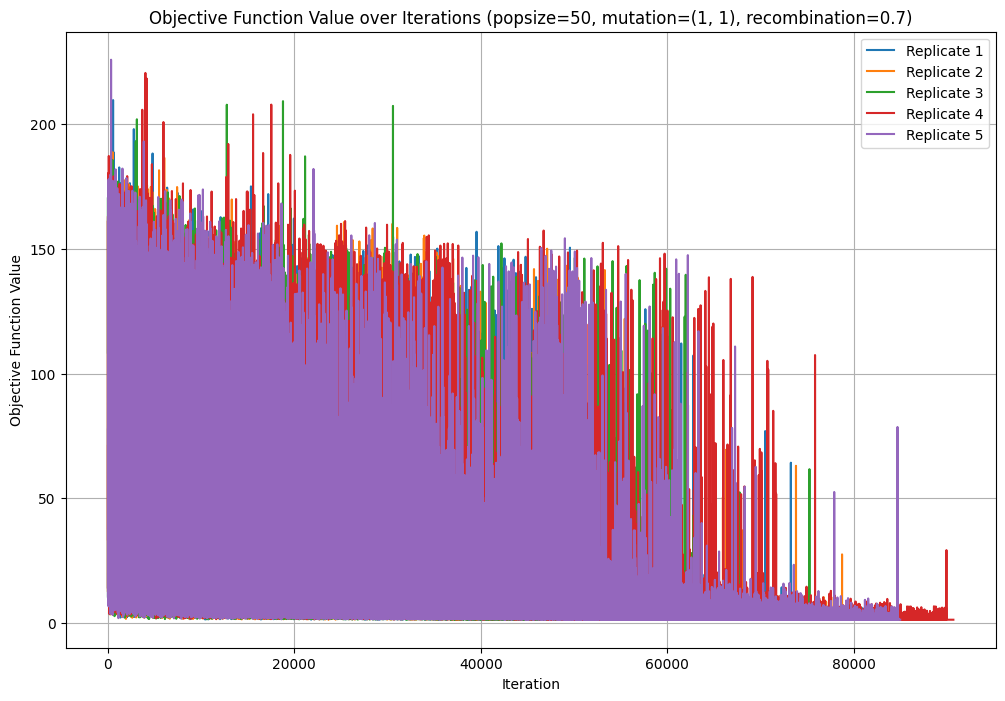 | 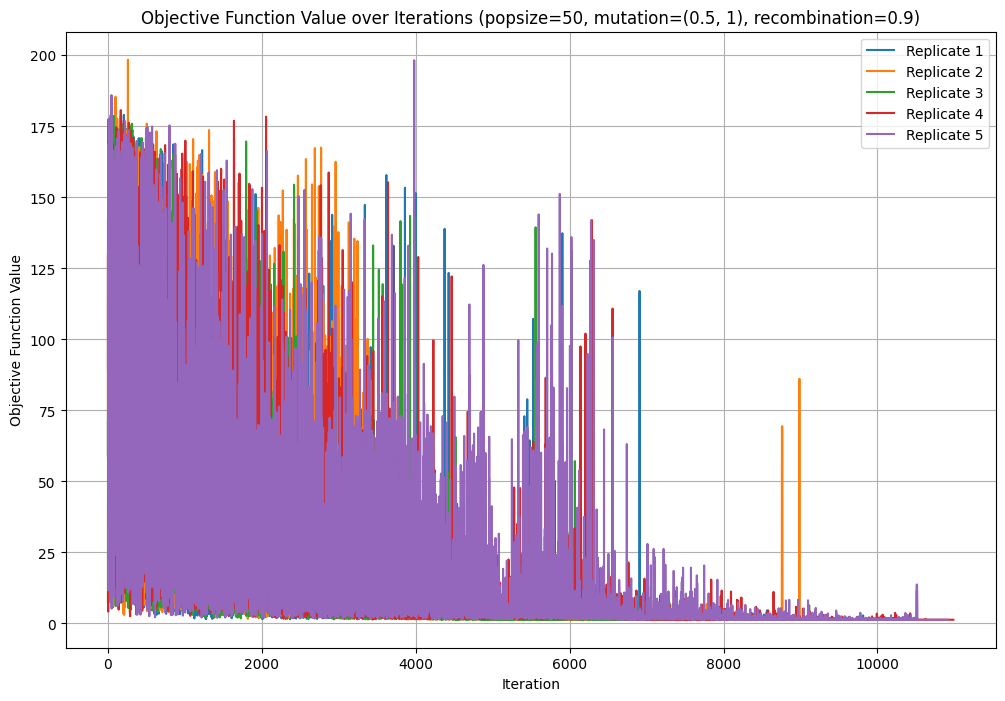 |

**Fig. 2**. Plot showing the number of iterations for convergence to the minimum value of the objective function by the differential evolution algorithm with varying hyperparameter configurations. Five replicate runs with varying random seeds were performed for each of the hyperparameter combinations tested.


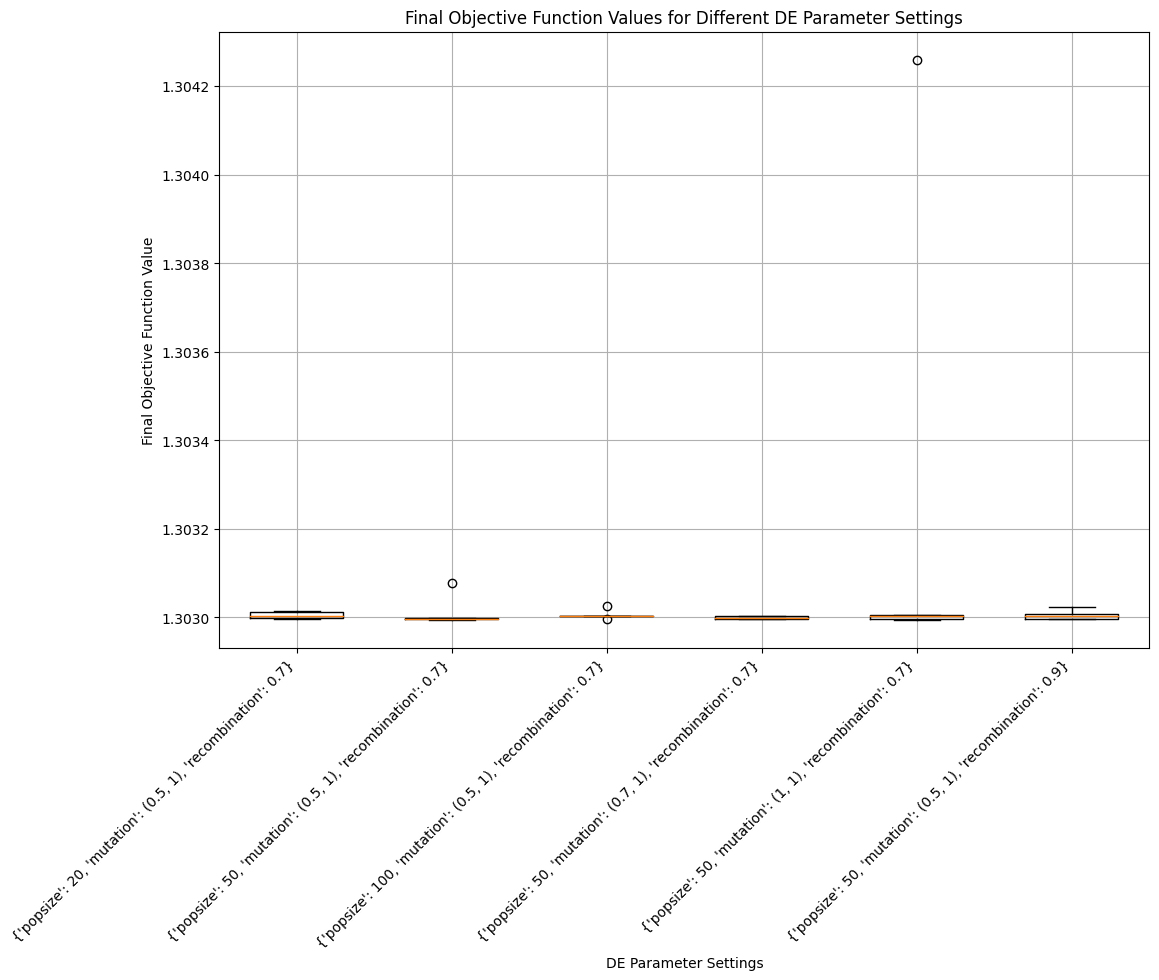


**Fig. 3.** Final objective function values obtained by the differential evolution algorithm for each of the hyperparameter combinations tested.

# **Pellicle formation in plate wells**

**
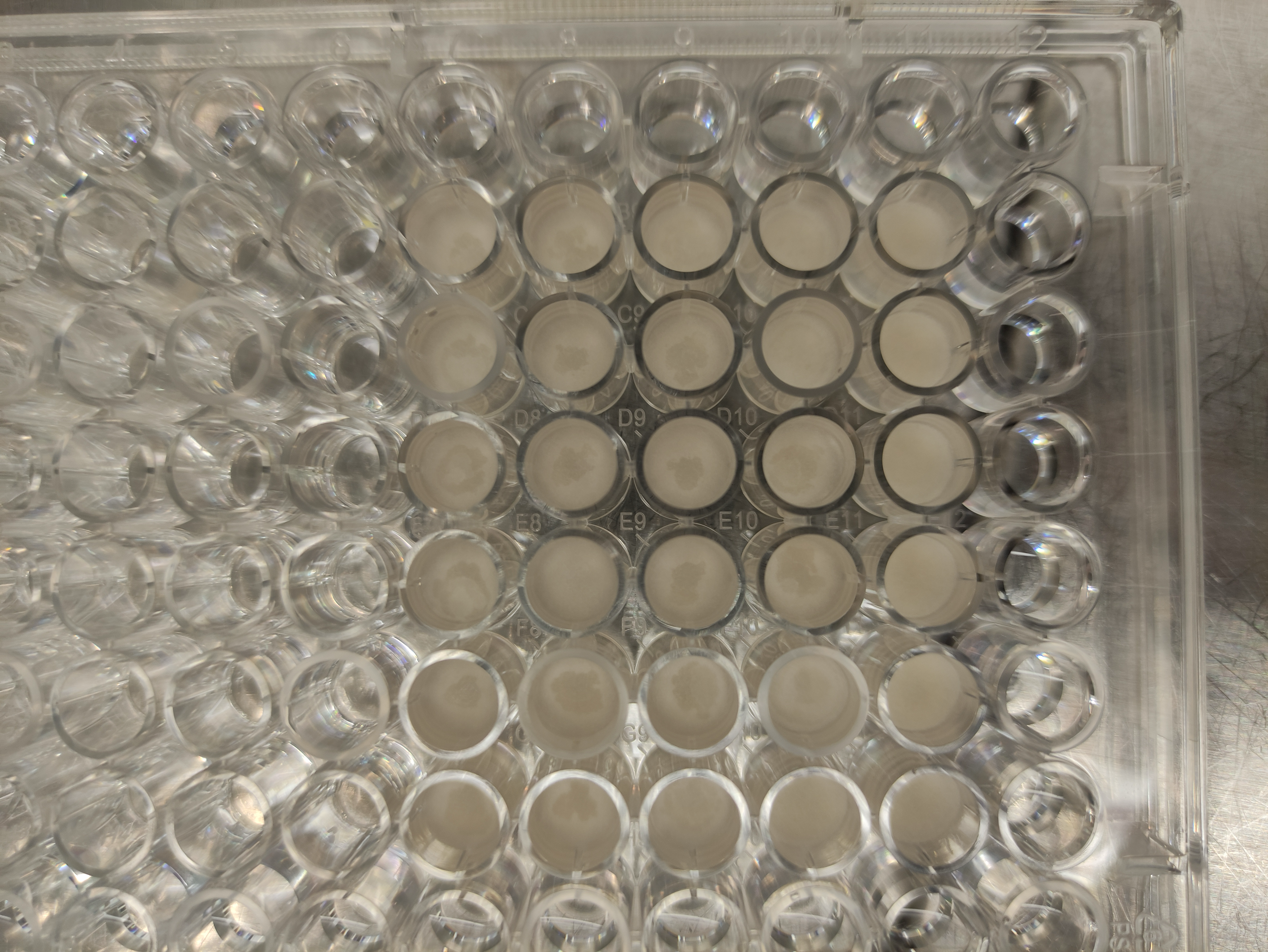
**

**Fig. 4**. Pellicle formation at the air-liquid interface of microplate wells during after 69.3 h of fermentation.

# **Parameter correlations and p-values**

**Table 4**. Correlation coefficients and p-values for pairwise parameters

| Parameter 1 | Parameter 2 | Correlation coefficient ($\rho$) | p-value (p) |
| --- | --- | --- | --- |
| $\mu_{m1}$ | $K_{c1}$ | 0.42 | 7.4e-66 |
| $\mu_{m1}$ | $\mu_{m2}$ | -0.32 | 4.1e-37 |
| $\mu_{m1}$ | $K_{c2}$ | -0.21 | 1.5e-16 |
| $\mu_{m1}$ | $k_{I}$ | 0.06 | 0.018 |
| $\mu_{m1}$ | $Y_{S1}$ | -0.34 | 1.3e-41 |
| $\mu_{m1}$ | $Y_{S2}$ | 0.21 | 4.7e-16 |
| $K_{c1}$ | $\mu_{m2}$ | 0.27 | 2.4e-27 |
| $K_{c1}$ | $K_{c2}$ | 0.23 | 9.4e-20 |
| $K_{c1}$ | $k_{I}$ | 0.75 | 1.2e-268 |
| $K_{c1}$ | $Y_{S1}$ | 0.14 | 3.3e-08 |
| $K_{c1}$ | $Y_{S2}$ | -0.03 | 0.28 |
| $\mu_{m2}$ | $K_{c2}$ | 0.69 | 1.4e-216 |
| $\mu_{m2}$ | $k_{I}$ | 0.46 | 1.2e-80 |
| $\mu_{m2}$ | $Y_{S1}$ | 0.8 | 0 |
| $\mu_{m2}$ | $Y_{S2}$ | -0.65 | 7.5e-183 |
| $K_{c2}$ | $k_{I}$ | 0.49 | 1.9e-92 |
| $K_{c2}$ | $Y_{S1}$ | 0.42 | 1e-64 |
| $K_{c2}$ | $Y_{S2}$ | -0.06 | 0.019 |
| $k_{I}$ | $Y_{S1}$ | 0.11 | 3.3e-05 |
| $k_{I}$ | $Y_{S1}$ | 0.08 | 0.001 |
| $Y_{S1}$ | $Y_{S2}$ | -0.73 | 3.3e-250 |

# **Contour plot of objective function with covariation of** $\boldsymbol{K}_{\boldsymbol{c1}}$ **and** $\boldsymbol{K}_{\boldsymbol{c}\boldsymbol{2}}$


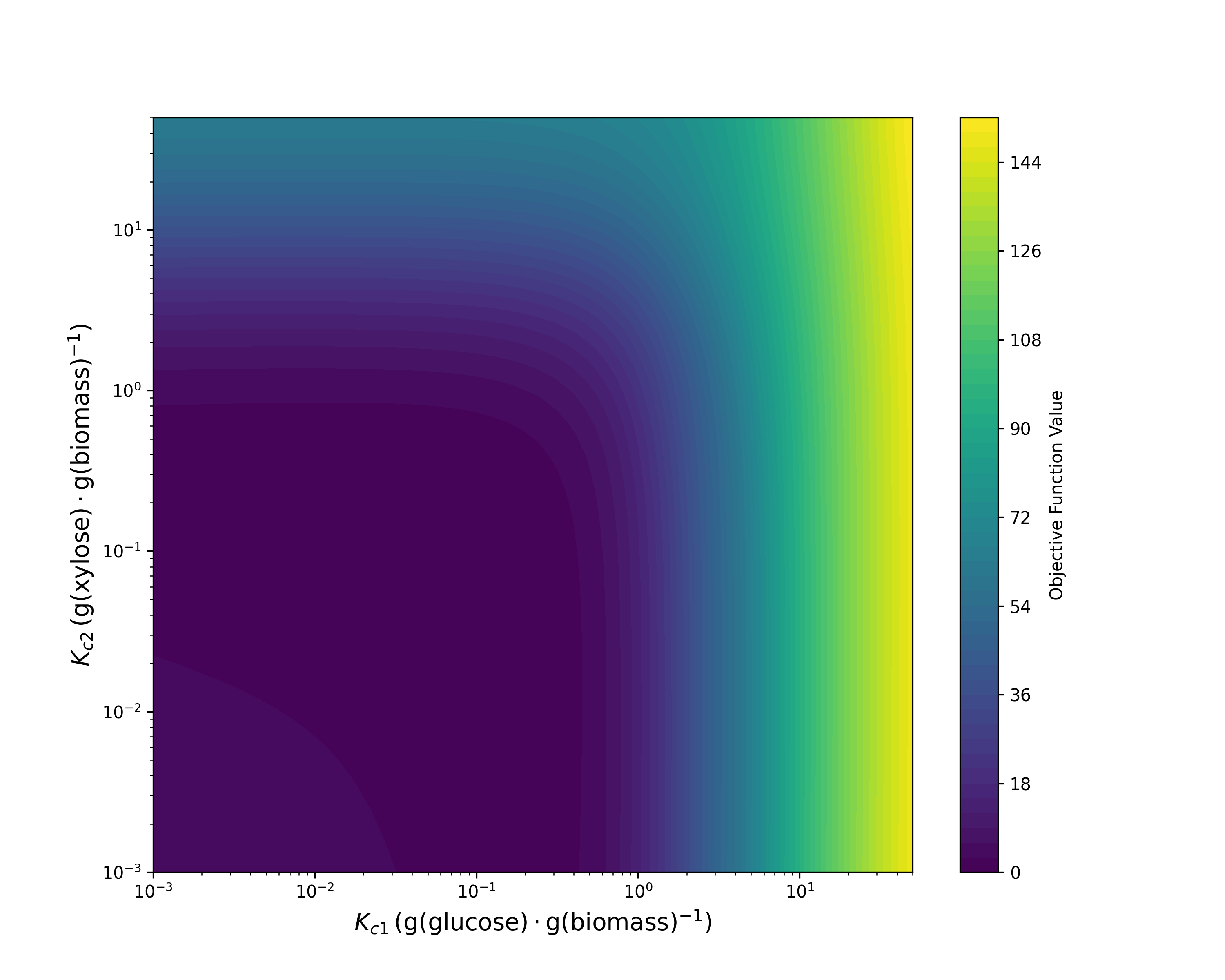


**Fig. 5**. The effect of covariation of $K_{c1}$and $K_{c2}$ on the value of the objective function. The large region of low sensitivity (dark blue) indicates a wide range of explainable parameter values for the current dataset.
